# Is something hidden during tachycardia?

**DOI:** 10.1007/s12471-016-0889-4

**Published:** 2016-09-01

**Authors:** D. Mol, M. T. Rijnierse, G. S. de Ruiter

**Affiliations:** 1 Department of Cardiology, OLVG, Amsterdam, The Netherlands; 2 Department of Cardiology, VU University Medical Center, Amsterdam, The Netherlands

**Fig. 1:**
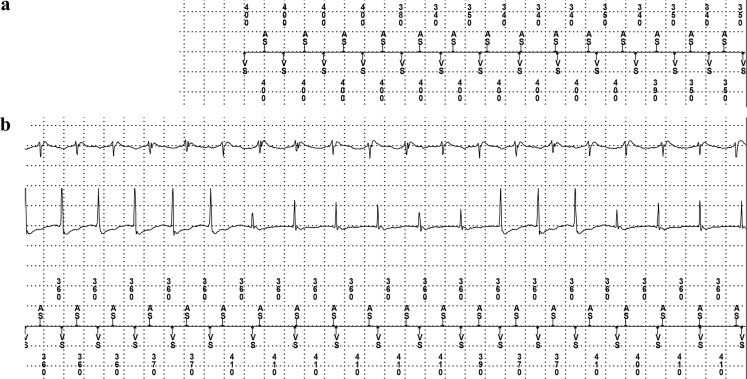
Intracardiac ICD recording prior to VT ablation; Classified as supraventricular tachycardia

A 61-year-old male patient was referred to our electrophysiology lab for ventricular tachycardia (VT) ablation. The patient had a history of ischaemic cardiomyopathy with impaired left ventricular function and received a dual chamber implantable cardioverter-defibrillator (ICD) after he survived a cardiac arrest. During the past months, the patient suffered from recurrent episodes of monomorphic VT with appropriate ICD therapies. Electrocardiogram suggested an apical origin of the VT. Prior to VT ablation, device interrogation showed a stored supraventricular tracing (Fig. 1).

**Question:** Is the device classification appropriate?

**Answer:** You will find the answer elsewhere in this issue.

